# Supplementary material for: Relationship Between Clusters of Multimorbidity and Dementia Risk: A Systematic Review
Source: Int J Geriatr Psychiatry. 2025 Sep 24;40(9):e70158. doi: 10.1002/gps.70158 (PMC12458968; doi:10.1002/gps.70158)
Supplement: Supplementary file 1 — Supporting Information S1 [file GPS-40-e70158-s002.docx]

**Electronic Literature Search of Embase, Ovid MEDLINE, and PsycINFO through Ovid**

| **Database** | **Steps** | **Search Terms** |
| --- | --- | --- |
| Embase,  Ovid MEDLINE,  PsycINFO | 1 | exp multimorbidity/** or exp comorbidity/ or exp “multiple chronic conditions”/** or multimorbidit* or comorbidit* or co-morbidit* or polymorbidit* or “patient complexit*” |
|  | 2 | exp dementia/ or dementia or alzheimer* disease or AD |
|  | 3 | exp Risk/** or exp Incidence/** or inciden* or risk* |
|  | 4 | Exp Cluster Analysis/ or cluster* or cluster analys* or pattern* or factor analys* |
|  | 5 | 1 and 2 and 3 and 4 |
|  | 6 | 5 not (exp qualitative research/ or exp case report/ or exp case series/ or exp review/ or exp conference abstract/ or exp book chapter/ or exp editorial/ or exp letter/) |
|  | 7 | limit 6 to (adult <18 to 64 years> or aged <65+ years>) *** |
|  | 8 | limit 7 to English language |
|  | 9 | remove duplicates from 8 |

Note. Different definitions of morbidity and comorbidity were identified through a scoping review (Dunn et al., 2022), highlighting the heterogeneity in how these concepts are operationalized. A comprehensive search was conducted using the Ovid interface to access Embase, Ovid MEDLINE, and PsycINFO databases. To ensure an inclusive and exhaustive search strategy, the term finder function was utilized to identify and incorporate relevant Emtree terms (specific to Embase), MeSH terms (specific to Ovid MEDLINE), and Thesaurus terms (specific to PsycINFO). *truncation. **the subject heading is invalid in APA PsycInfo. ***only works for Embase. Additional studies that are not eligible were removed in Rayyan
