# Supplementary material for: Relationship Between Clusters of Multimorbidity and Dementia Risk: A Systematic Review
Source: Int J Geriatr Psychiatry. 2025 Sep 24;40(9):e70158. doi: 10.1002/gps.70158 (PMC12458968; doi:10.1002/gps.70158)
Supplement: Supplementary file 4 — Supporting Information S4 [file GPS-40-e70158-s001.docx]

**Quality in Prognostic Studies (QUIPS) Tool Assessment**

| **Authors** | **Study Participation** | **Study Attrition** | **Prognostic Factor Measurement** | **Outcome Measurement** | **Study Confounding** | **Statistical Analysis and Reporting** |
| --- | --- | --- | --- | --- | --- | --- |
| Calvin et al., 2022 | Low | High | Low | Moderate | Low | Low |
| Grande et al., 2021 | Low | Moderate | Low | Low | Low | Low |
| Hu et al., 2022 | Low | High | Low | Moderate | Low | Low |
| Khondoker et al., 2023 | Low | High | Low | Moderate | Low | Low |
| Patel et al., 2024 | Low | High | Low | Moderate | Moderate | Low |
| Valletta et al., 2023 | Low | High | Low | Low | Low | Low |
| Wang et al., 2024 | Low | High | Low | Moderate | Low | Low |

Note. Low = low risk of bias; Moderate = moderate risk of bias; High = high risk of bias.
